# Supplementary material for: Molecular Dynamics Study of the Curvature-Driven Interactions between Carbon-Based Nanoparticles and Amino Acids
Source: Molecules. 2023 Jan 4;28(2):482. doi: 10.3390/molecules28020482 (PMC9861389; doi:10.3390/molecules28020482)
Supplement: Supplementary file 1 [file molecules-28-00482-s001.zip › molecules-2125350-supplementary.pdf]

Supplementary Information for

Molecular Dynamics Study of the Curvature-Driven Interactions between Carbon-Based Nano Particles and Amino Acids

Wanying Huang [1], Zhenyu Wang [2] and JunYan Luo [3,*]

[1] T-Life Research Center, State Key Laboratory of Surface Physics, Department of Physics, Fudan University, Shanghai 200433, China
[1] Zhejiang Lab, Nanhu-Kechuang Avenue, Yuhang District, Hangzhou 310000, China
[2] T-Life Research Center, State Key Laboratory of Surface Physics, Department of Physics, Fudan University, Shanghai 200433, China
[3] Department of Physics, Zhejiang University of Science and Technology, Hangzhou 310023, China
* Correspondence: jyluo@zust.edu.cn

From CNT55L3, CNT1010L3, CNT1515L3 to CNT2020L3, as shown in Figure S1A, the radii of carbon nanotubes with different radii increase, and the curvature decreases. The parameters of each carbon-based nanoparticle are shown in Figure S1D-Table. In our research, to exclude the possibility of amino acids entering the carbon nanotubes as the CNT radius increases sequentially, we have used periodic boundary conditions. In other words, we studied the periodic infinitely long carbon tube CNT and the carbon-based plane Graphene, which allows us to focus only on the effect of the curvature of the carbon-based nanoparticle on its adsorption effect.

In addition, in order to eliminate the influence of CBNs volume on the adsorption effect, when constructing the system, we followed the principle of the same surface contact volume ratio of all simulation systems, namely

$$\varphi = \frac{S_{CBNs}}{V - V_{CBNs}}$$

The φ value of all simulation systems is fixed, that is, the surface entropy of the adsorption space of the system is a fixed value. Among them, S_{CBNs} represents the surface area of carbon-based nanoparticles, V represents the volume of the system, and V_{CBNs} represents the volume of carbon-based nanoparticles.

A

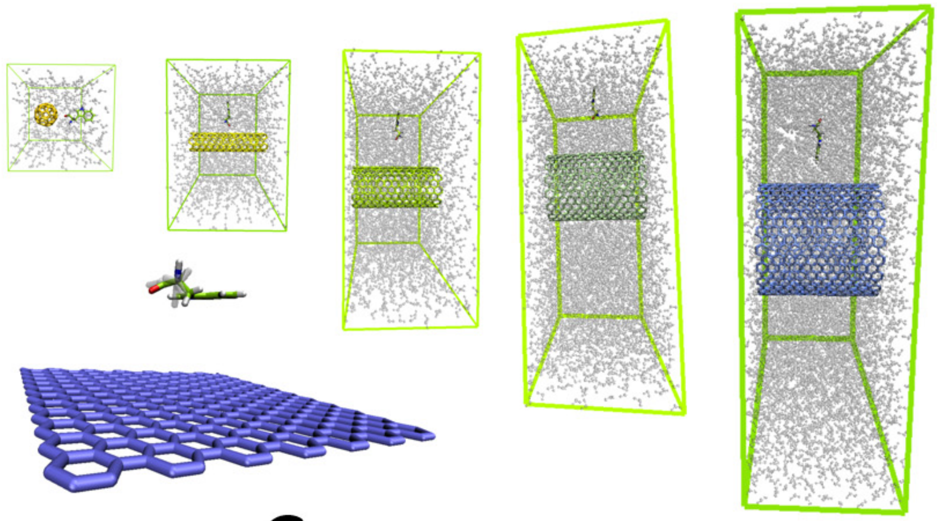

B

Group	Animoacids
Nonpolar	1-Gy
	2-Ala
	3-Pro
	4-Val
	5-Leu
	6-Ile
	7-Met
Polar	8-Ser
	9-Thr
	10-Cys
	11-Asn
	12-Gln
Aromatic	13-Phe
	14-Tyr
	15-Trp
Positively	16-Lys
	17-Arg
	18-His
Negatively	19-Asp
	20-Glu

C

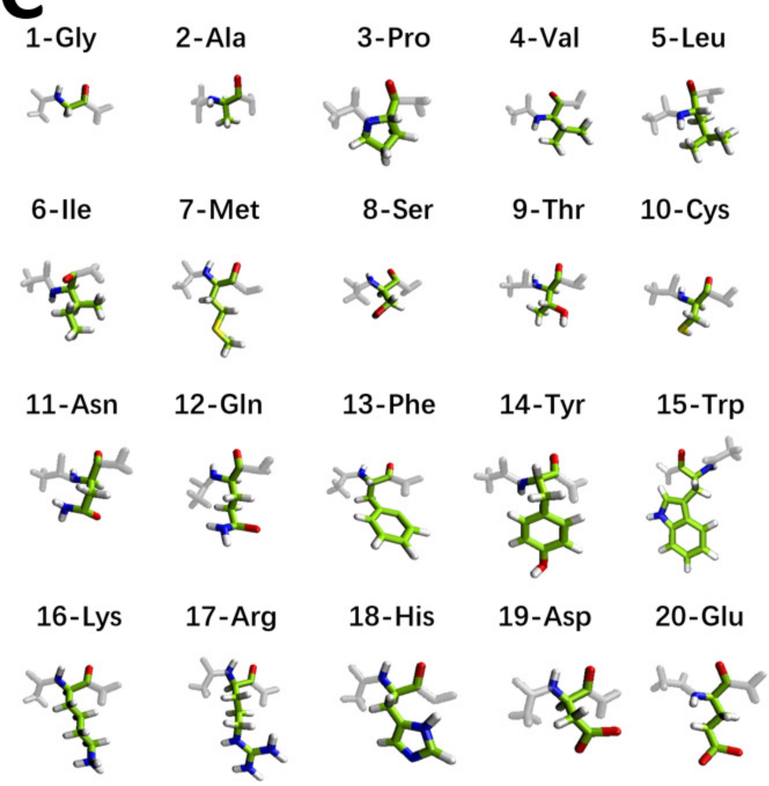

D

Number	CBNs	Lx(nm)	Ly(nm)	Lz(nm)	diameter	Radius(nm)	Curvature(1/nm)	S/(V-Vcnt)
C1	C60	2.25	2.25	2.25	0.668 nm	0.334	2.99401	0.125
C2	CNT55L3	4.16	4.16	2.93	0.673 nm	0.3365	2.97177	0.125
C3	CNT1010L3	5.94	5.94	2.93	1.346 nm	0.673	1.48588	0.125
C4	CNT1515L3	7.36	7.36	2.93	2.020 nm	1.01	0.99009	0.125
C5	CNT2020L3	8.61	8.61	2.93	2.712 nm	1.356	0.73746	0.125
C6	Graphene33	2.95141	2.982	8.22	3 nm×3nm	→ ∞	→ 0	0.125

Figure S1. A. Schematic diagram of amino acid and carbon-based nanoparticle simulation system (take 15-TRP as an example): orange is C60, yellow is CNT55L3, light green is CNT1010L3, green is CNT1515L3, bright blue is CNT2020L3, and

purple is Graphene33. B. The Group of 20 amino acids: the orange component is the nonpolar amino acid group; the yellow component is the polar uncharged amino acid group; the green component is the aromatic amino acid group; the blue component is the positively charged amino acid group; the purple components is negatively charged amino acids. C. Schematic diagram of the molecular structure of 20 amino acids. D. Table: the spatial size list of six representative carbon nanoparticles and amino acid systems

Our research, based on previous studies of molecular dynamics between carbon-based nanoparticles and proteins by other researchers, has grouped 20 amino acids. As shown in Figure S1B, they are (1) Nonpolar amino acids (Nonpolar, aliphatic R group):1-Gly, 2-Ala, 3-Pro, 4-Val, 5-Leu, 6-Ile, 7-Met; (2) Polar amino acids (Polar, uncharged R group): 8-Ser, 9-Thr, 10-Cys, 11-Asn, 12-Gln; (3) Aromatic amino acids (Aromatic R group): 13-Phe, 14-Tyr, 15-Trp; (4) Positively charged amino acids (Positively charged R group): 16-Lys, 17-Arg, 18-His; (5) negatively charged amino acids (Negatively charged R group): 19-Asp, 20-Glu. Such a combination arrangement allows us to study well, which properties of the amino acid side chain affect its adsorption effect with carbon-based nanoparticles.

The molecular structures of 20 amino acids are shown in Figure S1C. Among them, the amino acids of the non-polar amino acid group start from amino acids 1-7, and the side chains increase sequentially. The shortest side chain is 1-Gly, and the longest is 7-Met; the amino acids of the polar non-charged amino acid group are from 8-12 In the positively charged amino acid group, we put 18-His with a benzene ring at the end, but the 17-Arg in this group is the positively charged amino acid with the longest side chain.

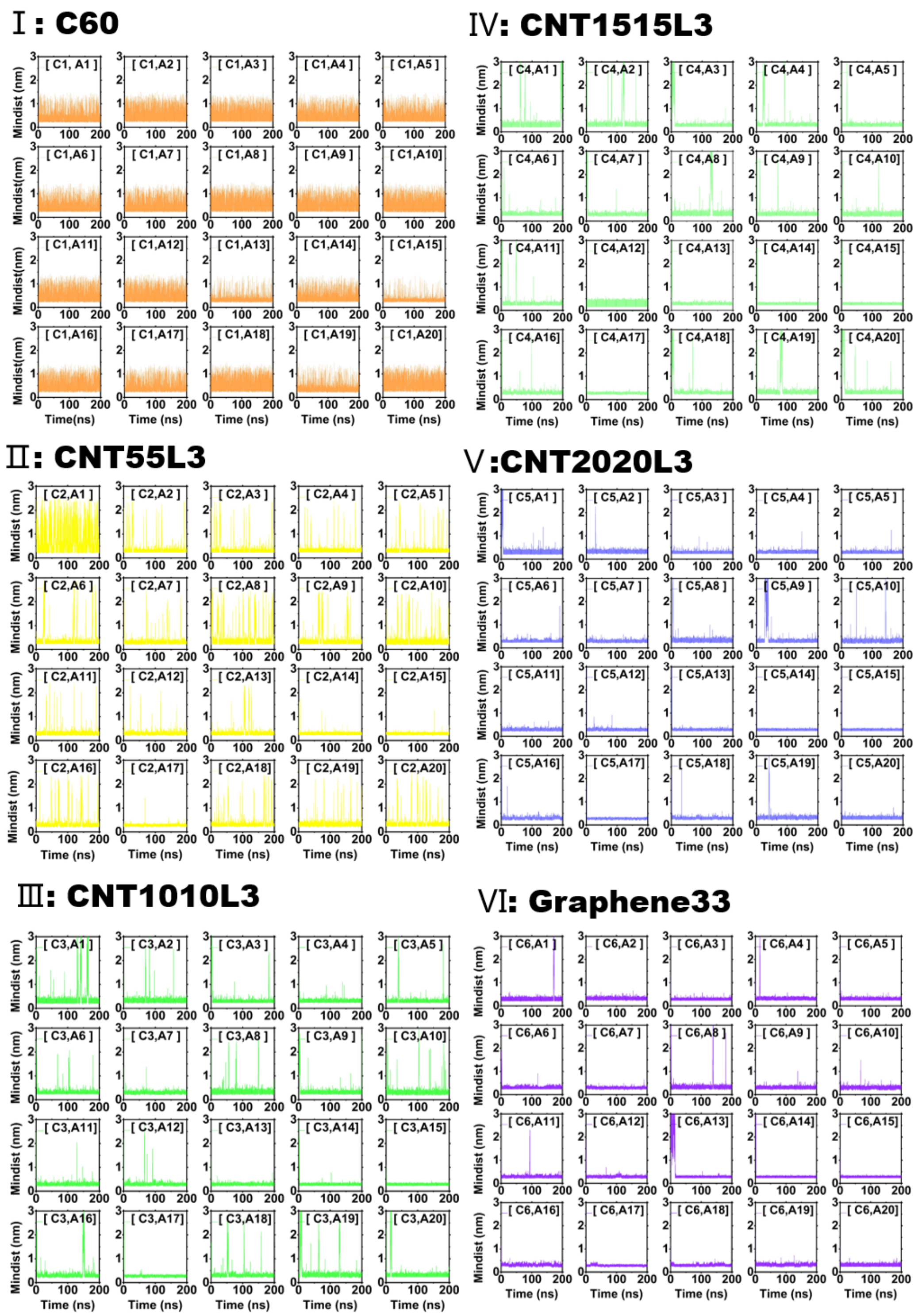

Figure S2. The change of the minimum distance between CBNs and amino acids over time, the horizontal axis t (ns), the vertical axis Mindist (nm). From C1-C6 respectively: I group picture C1 orange line C60, group II picture C2 yellow line CNT55L3, group III picture C3 bright green line CNT1010L3, group IV picture C4 green line CNT1515L3, group V picture C5 bright blue line CNT2020L3, VI Group picture C6 purple line Graphene33; each group picture contains 20 kinds of representative amino acids and Mindist changes over time between CBNs, non-polar amino acid group A1-

A7 are: 1-Gly, 2-Ala, 3-Pro, 4-Val, 5-Leu, 6-Ile, 7-Met; Polar uncharged amino acid group A8-A12 are: 8-Ser, 9-Thr, 10-Cys, 11-Asn, 12-Gln; Aromatic amino acid group A13-A15 are: 13-Phe, 14-Tyr, 15-Trp; Positively charged amino acid group A16-A18 are: 16-Lys, 17-Arg, 18-His; Negatively charged amino acid group A19-A20 are: 19-Asp, 20-Glu.

We could observe the dynamic changes from the perspective of the curvature of carbon-based nanoparticles in Figure S2. Figure S2I group picture C1 orange line C60, the adsorption performance of 20 representative amino acids and C60 is not good, most of them are in the state of pulsating separation in space; Figure S2II group picture C2 yellow line CNT55L3, most of the adsorption effect with CNT55L3 is better than that of C60 group There was a short stay; Figure S2III group C3 bright green line CNT1010L3, some amino acids showed strong adsorption performance, especially aromatic amino acids; Figure S2IV group C4 green line CNT1515L3, the overall amino acid adsorption effect is not bad, even 5-Leu and CNT1515L3 both showed good adsorption performance; Figure S2V group picture C5 bright blue line CNT2020L3, most of the amino acids have excellent adsorption effects, except for a few amino acids, such as 9-Thr and CNT2020L3, part of the separation occurs; Figure S2VI Group Graph C6 Purple line Graphene33, this is the group with the best overall adsorption effect, and some short-term "jumping" behaviors can also be ignored.

We indeed observe the changes in the adsorption effect of carbon-based nanoparticles with 20 amino acids from the perspective of changes in the curvature of carbon-based nanoparticles, and some trends can be preliminarily drawn, but rigorous analysis and judgment still require statistical research.

（a）Mindist-distribution between CBNs and 20 amino acids

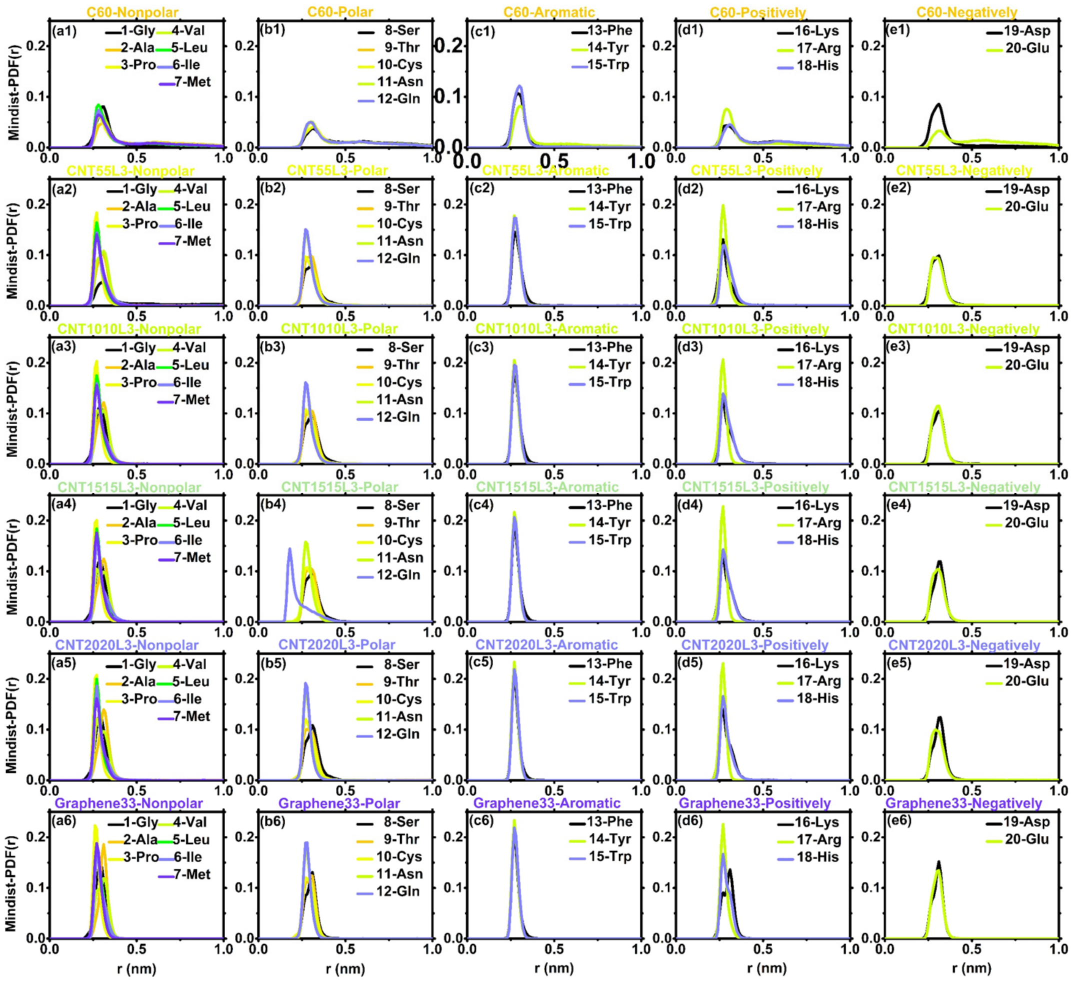

Figure S3. The mindist distribution between 20 kinds of amino acids and 6 kinds of carbon-based nanoparticles. Numbers 1-6 are each group of amino acids and 6 kinds of carbon-based nanoparticles 1-C60, 2-CNT55L3, 3 -CNT1010L3, 4-CNT1515L3, 5-CNT2020L3, 6-Graphene33, the amino acid groups are: (a1)-(a6) are nonpolar amino acid group, the black line is 1-Gly, the orange line It is 2-Ala, the yellow line is 3-Pro, the light green line is 4-Val, the bright green line is 5-Leu, the blue line is 6-Ile, and the purple line is 7-Met; (b1)-(b6) is Polar amino acid group, the black line is 8-Ser, the orange line is 9-Thr, the yellow line is 10-Cys, the light green line is 11-Asn, and the blue line is 12-Glu; (c1)-(c6) is the Aromatic amino acid group, the black line is 13-Phe, the light green line is 14-Tyr, the blue line is 15-Trp; (d1)-(d6) is the positive Positively charged amino acid group, the black line is 16-Lys, the light green line is 17-Arg, the blue line is 18-His; (e1)-(e6) are negatively charged amino acid group, The black line is 19-Asp, and the light green line is 20-Glu.

We will analyze the statistical distribution from the data that the minimum distance changes over time. This research step is very necessary because, in Mindist data, some data smaller than 0.3 nm are actually in dynamic changes. As shown in Figure S3, we can macroscopically observe the adsorption relationship between each group of amino acids and CBNs. Among them, the adsorption effect of aromatic amino acids and CBNs is the best, and their First-peak can be greater than 0.225, while the first-peak of negatively charged amino acids hovers around 0.1, and the overall is less than 0.15. Non-polar amino acids have different adsorption strengths from CBNs according to their side chains and chain lengths; polar non-charged amino acids have different internals, but the overall adsorption strength is lower than non-polar amino acids; The strongest positively charged amino acids are comparable to aromatic amino acids, But the overall adsorption strength of negatively charged amino acids is weak.

（b）The adsorption between CBNs and 20 amino acids

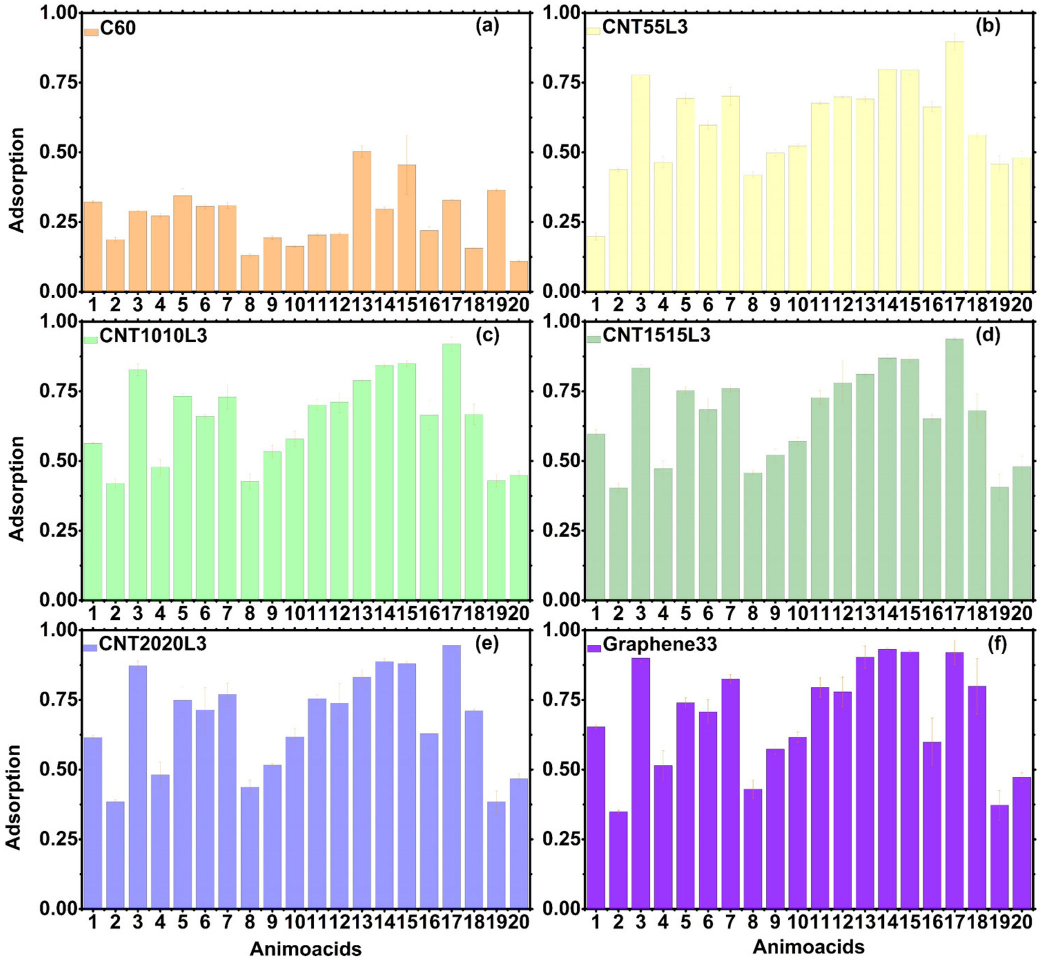

Figure S4. The statistics of the adsorption distance of various carbon-based

nanoparticles for 20 amino acids less than 0.3nm: (a) the average adsorption statistics of 20 amino acids by C60. (b) the average adsorption statistics of 20 amino acids by CNT55L3, (c) the average adsorption statistics of 20 amino acids by CNT1010L3. (d) the average adsorption statistics of 20 amino acids by CNT1515L3. (e) the average adsorption statistics of 20 amino acids by CNT2020L3, and (f) the average adsorption statistics of 20 amino acids by Graphene33 Adsorption statistics.

The adsorption strength with 0.3 nm. In Figure S4, we tried to count the changes in the overall adsorption strength of different amino acids when the minimum distance between CBNs and 20 amino acids was less than 0.3nm. We ranked each group of amino acids. Among them, we set different color settings for the adsorption statistics of different carbon-based nanoparticles. We can see that, as shown in Figure S4, the adsorption strength between the black C60 and each amino acid is the lowest overall, while the other groups have little difference. Among them, the order of the Nonpola part in Figure S4-b(c)(d)(e) part is the same, all of which are 2-Ala, 4-Val, 6-Ile, 5-Leu, 7-Met, and 3-Pro. But it seems that this classic statistical method does not have too many regular results for the adsorption of individual amino acids to carbon-based nanoparticles. However, it is worth mentioning that 3-Pro, a “benzene-like” amino acid, shows excellent adsorption capacity with CBNs.

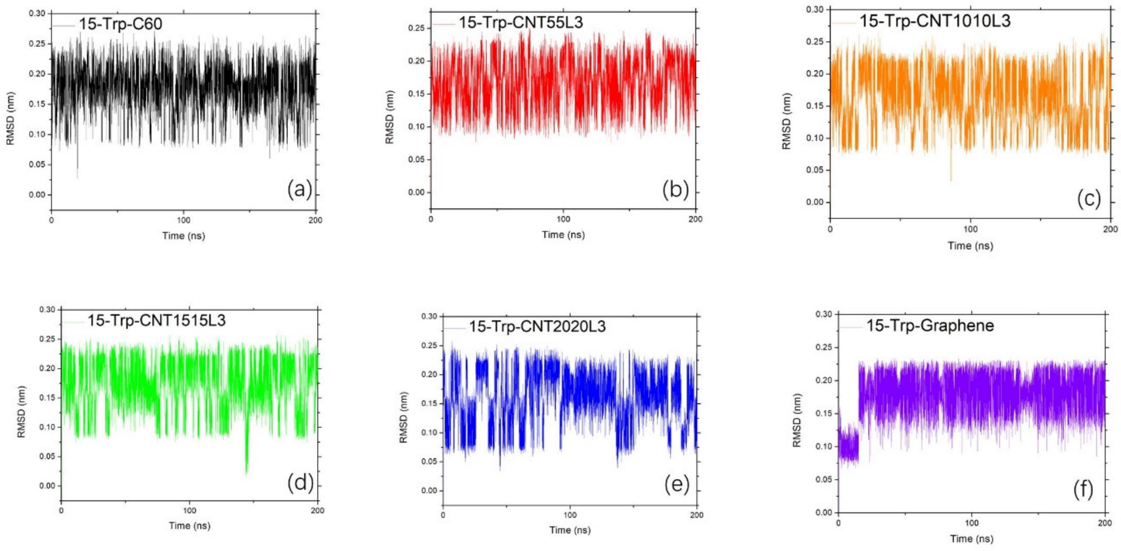

Figure S5. The Changing Trend of RMSD of Aromatic Amino Acids 15-Trp with Time: (a) black line for Trp with C60; (b) red line for Trp with CNT55L3; (c) orange line for Trp with CNT1010L3; (d) green line for Trp with CNT1515L3; (e) blue line for Trp with CNT2020L3.

The changing trend of RMSD of aromatic amino acids with time. When 15-Trp interacts with different carbon-based nanoparticles, the numerical range of RMSD is obviously different. It is worth noting that the better the adsorption effect is, the more periodic the RMSD value of 15-Trp will show.
